# Selective Interaction of a Cationic Polyfluorene with Model Lipid Membranes: Anionic *versus* Zwitterionic Lipids

**DOI:** 10.3390/ma7032120

**Published:** 2014-03-13

**Authors:** Zehra Kahveci, María José Martínez-Tomé, Rocío Esquembre, Ricardo Mallavia, C. Reyes Mateo

**Affiliations:** Instituto de Biología Molecular y Celular, Universidad Miguel Hernández, Elche (Alicante) 03202, Spain; E-Mails: zkahveci@umh.es (Z.K.); mj.martinez@umh.es (M.J.M.-T.); resquembre@umh.es (R.E.); r.mallavia@umh.es (R.M.)

**Keywords:** polyelectrolyte, polyfluorene, fluorescent membrane marker, liposomes, bioimaging

## Abstract

This paper explores the interaction mechanism between the conjugated polyelectrolyte {[9,9-bis(6’-N,N,N-trimethylammonium)hexyl]fluorene-phenylene}bromide (HTMA-PFP) and model lipid membranes. The study was carried out using different biophysical techniques, mainly fluorescence spectroscopy and microscopy. Results show that despite the preferential interaction of HTMA-PFP with anionic lipids, HTMA-PFP shows affinity for zwitterionic lipids; although the interaction mechanism is different as well as HTMA-PFP’s final membrane location. Whilst the polyelectrolyte is embedded within the lipid bilayer in the anionic membrane, it remains close to the surface, forming aggregates that are sensitive to the physical state of the lipid bilayer in the zwitterionic system. The different interaction mechanism is reflected in the polyelectrolyte fluorescence spectrum, since the maximum shifts to longer wavelengths in the zwitterionic system. The intrinsic fluorescence of HTMA-PFP was used to visualize the interaction between polymer and vesicles via fluorescence microscopy, thanks to its high quantum yield and photostability. This technique allows the selectivity of the polyelectrolyte and higher affinity for anionic membranes to be observed. The results confirmed the appropriateness of using HTMA-PFP as a membrane fluorescent marker and suggest that, given its different behaviour towards anionic and zwitterionic membranes, HTMA-PFP could be used for selective recognition and imaging of bacteria over mammalian cells.

## 1. Introduction

Polyelectrolytes are polymers of any type and structure that carry positively or negatively charged ionizable groups. The interaction of these macromolecules with lipids or cell membranes play an important role in many biophysical applications, such as a safe and targeted delivery of genetic material to cells, stabilization of liposomes, or antimicrobial activity [1–5]. Conjugated polyelectrolytes (CPEs) are a particular type of polyelectrolytes, which have very interesting properties. They are polymers with π-conjugated backbones, which show strong absorption and high efficiencies in both photoluminescence and electroluminescence, and contain ionic side groups to facilitate their water solubilisation [6,7]. Over the past decades, CPEs have received a lot of attention in biomedical applications, especially for developing biosensing schemes and sensing devices for biomolecules [8–11], and more recently they have been employed as novel fluorescent probes for bioimaging [12–19]. Among CPEs, those involving fluorene-based systems offer the advantage of high fluorescence quantum yields and photostability, blue emission (suitable for energy transfer experiments), excellent thermal stability and high stability against oxidants, as well as good synthetic accessibility [19,20]. In addition, fluorene-based CPEs consist of a rigid hydrophobic polyfluorene backbone with flexible charged side chains, which induces interesting aggregation behaviour; this phenomenon directly affects their intrinsic fluorescence [21–24]. These properties have been used for studying interactions with biomolecules, such as proteins and DNA, allowing sensing platforms to be developed. However, the interaction of charged polyfluorenes with model lipid membranes, which imitate the complexity of natural cell membranes, still remains practically unexplored [25–27]. This study is of interest for predicting and understanding the mechanism of interaction with real cell membranes in order to develop new biotechnological applications.

**Figure 1.**
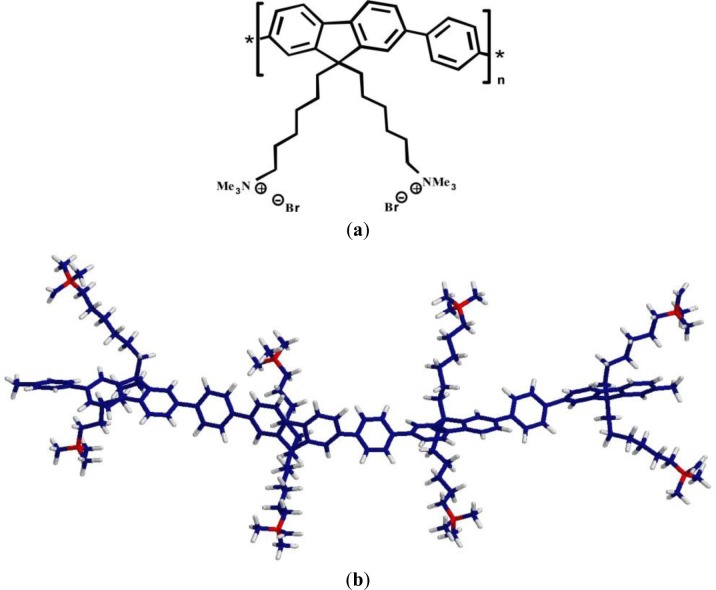
(**a**) HTMA-PFP chemical structure; (**b**) the conformation of a tetramer of HTMA-PFP obtained using molecular dynamics simulations.

The cationic polyelectrolyte poly{[9,9-bis(6’-N,N,N-trimethylammonium)hexyl]fluorene-phenylene} bromide (HTMA-PFP) is a water-soluble polyfluorene, consisting of a backbone and alkyl side chain hydrophobic moieties, while the cationic charged quaternary amines control electrostatic interactions (Figure 1) [28,29]. Due to this structure, the polyelectrolyte tends to establish nonspecific electrostatic interactions with species of opposite charge, such as DNA or proteins like human serum albumine (HSA), which can be monitored from changes in its absorption and intrinsic fluorescence [25,27]. In addition, the polyelectrolyte is a good energy transfer donor for acceptors over a large part of the visible spectrum because of its high fluorescent quantum yield and blue emission, and it has also been shown to be an adequate energy acceptor from tryptophan residues in peptides and proteins [25,30]. Recently, we explored the interaction of HTMA-PFP with vesicles composed of anionic phospholipids [31]. In that study, we concluded that the polyelectrolyte rapidly incorporated into the membrane with high affinity, being embedded within the lipid bilayer where it showed high fluorescence efficiency and good stability. These properties allow the visualization of the lipid vesicles with fluorescence microscopy.

Fluorescent imaging of bacterial infection by selectively targeting the bacterial membrane over the membrane surfaces of healthy mammalian cells has emerged as a powerful tool with many health and environmental applications, such as visualizing small populations of bacterial cells in biological fluids or in food and drinks [32–35]. However, in spite of its advantages, to date only a small number of targeting probes have been reported for the aforementioned application. Ideal markers should be highly fluorescent, water-soluble, biocompatible, photostable, and composed of two structural components: an affinity ligand and a reporter group. The high affinity of HTMA-PFP for anionic lipids, the dominant lipid component in bacterial membranes, as well as its high fluorescence quantum yield and photostability makes HTMA-PFP a suitable candidate for this task. To explore this possibility, the selectivity of the polymer against model bacterial and mammalian membranes should be investigated. It is known that the mammalian plasma membrane is rich in zwitterionic lipids. By varying the lipid composition of the model membranes, it is possible to mimic both types of cell membranes. In this paper, we investigated the affinity and mechanism of HTMA-PFP interaction with membranes composed of zwitterionic phospholipids and the results were compared with those obtained in anionic membranes. In addition, we determined the behaviour of the polyelectrolyte in samples that simultaneously contained both anionic and zwitterionic vesicles in different ratios. Finally, it is known that molecules having quaternary amine groups are able to disrupt bacterial membranes causing cell death [36–38], and therefore, the membrane perturbing activity of HTMA-PFP was also evaluated.

## 2. Results and Discussion

### 2.1. HTMA-PFP in Zwitterionic Membranes

In a previous paper, we investigated the behaviour of HTMA-PFP in buffer and in the presence of vesicles composed of anionic phospholipids [31]. The polyelectrolyte showed a low fluorescence quantum yield and a red-shift of the emission spectrum in buffer, which was attributed to the formation of metastable aggregates by self-assembly of the polymer hydrophobic chains and to the existence of nonspecific electrostatic interactions between these aggregates and anionic species contained in the buffer. A strong increase in the fluorescence intensity and a blue-shift of the spectrum was observed in the presence of low concentrations of anionic vesicles, indicating that HTMA-PFP interacts with the lipid bilayer with high affinity and, as a consequence of this interaction, the polymer chains become more extended than in buffer and the probability of polymer-polymer interaction (aggregation) is reduced. Additional experiments showed that the polyelectrolyte penetrates into the hydrophobic core, labelling the lipid bilayer without altering the morphology of the vesicles and allowing their visualization.

**Figure 2.**
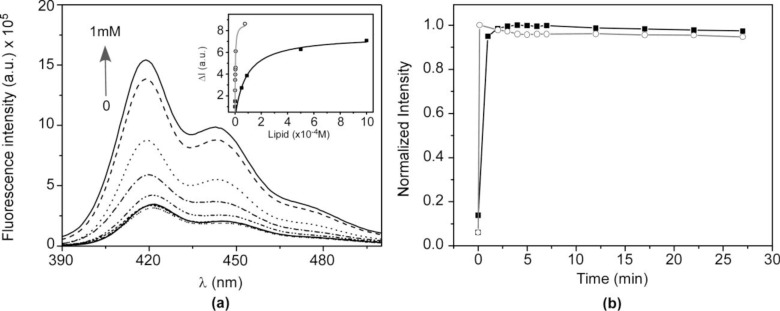
(**a**) Emission spectra recorded for HTMA-PFP (1.5 μM) at different DMPC concentrations 0, 0.001, 0.002, 0.003, 0.004, 0.005, 0.05, 0.085, 0.5, 1 mM. **Inset:** changes in fluorescence intensity (*∆I*) at increasing concentrations of DMPC (black line) and DMPG (gray line); (**b**) incorporation kinetics of HTMA-PFP in DMPC (black line) and DMPG (gray line) labelled with BODIPY 500/510 C_4_, C_9_ measured at 40 °C by monitoring the fluorescence intensity recorded at λ_em_ = 516 nm (λ_ex_ = 380 nm) after addition of polyelectrolyte.

The high affinity of HTMA-PFP to anionic phospholipids is probably due to the electrostatic interaction between the quaternary amine groups of HTMA-PFP and the negative charge of the lipid head groups. Therefore, it should be of interest to know how the polymer-membrane interaction is affected by the lipid charge in order to determine the membrane selectivity of HTMA-PFP and evaluate its potential use as a fluorescent probe for bacterial imaging. To this end, we studied the interaction mechanism of the polyelectrolyte with lipid vesicles composed of zwitterionic phospholipids, comparing the results with those obtained for anionic lipid vesicles. As a first step, the affinity of HTMA-PFP to zwitterionic membranes was evaluated in samples containing large unilamellar vesicles (LUVs) of 1,2-Dimyristoyl-*sn*-glycero-3-phosphocholine (DMPC). Samples were prepared with increasing lipid concentrations (up to 1 mM) and the same concentration of HTMA-PFP (1.5 μM, in terms of repeat units). The temperature was maintained at 40 °C to ensure that the lipid bilayer was in fluid phase (DMPC presents its main thermal phase transition around 24 °C). Figure 2a shows the emission spectra recorded for the different samples. An enhancement of the fluorescence intensity and a very small blue-shift in the spectrum was observed up to a lipid concentration of about 1 mM. Higher concentrations of lipid did not significantly modify the fluorescence signal, suggesting that, at this lipid concentration, all the polymer chains interacted with the lipids and no aggregates remained in the buffer. Changes in the fluorescence intensity were used to estimate the partition coefficient of the polyelectrolyte between the zwitterionic membrane and the aqueous phase (*K_P_*), which is defined in terms of molar concentrations as:

$$K_{P}=\frac{n_{L}/V_{L}}{n_{W}/V_{W}}$$ (1)

where *n_i_* stands for moles of compound in phase *i* and *V_i_* for volume of phase *i*. The phase is either aqueous (*i = W*) or lipidic (*i = L*). The quantitation of *K_P_* was performed according to [39]:

$$\Delta I=\frac{\Delta I_{\max}[L]}{\frac{1}{K_{P}\gamma }+[L]}$$ (2)

where *∆I* (*∆I = I* − *I*_0_) stands for the difference between the fluorescence intensity of HTMA-PFP measured in the presence (*I*) and in the absence (*I*_0_) of the phospholipid vesicles, *∆I*_max_ *= I*_∞_ − *I*_0_ is the maximum value of this difference once the limiting value is reached (*I*_∞_) upon increasing the phospholipid concentration [*L*], and γ is the molar volume of the phospholipid (for DMPC in the fluid phase the value of γ is 0.9 M^−1^) [40]. Comparing these results with those obtained for liposomes composed of the anionic phospholipid 1,2-Dimyristoyl-*sn*-glycero-3-phospho-rac-(1-glycerol) DMPG (see inset in Figure 2a), we conclude that HTMA-PFP shows higher affinity for anionic vesicles than for zwitterionic ones. In fact, the *K_P_* value obtained for DMPG in the previous paper was one order of magnitude higher than that for DMPC [31].

The incorporation kinetics of the polyelectrolyte into the zwitterionic membrane was explored by means of FRET experiments using BODIPY 500/510 C_4_, C_9_, which was previously shown to be a good energy acceptor of HTMA-PFP [31]. This BODIPY fatty acid was incorporated into the DMPC membrane before polymer addition, at a probe:lipid molar ratio of 1:500. Its fluorescence emission (λ_em_ = 516 nm) was practically negligible upon excitation at the absorption maximum wavelength of the polymer (380 nm), which was an expected result, since BODIPY does not absorb appreciably at this wavelength. HTMA-PFP’s interaction with the membrane was monitored by recording the increase in the fluorescence signal as a function of time, as a consequence of the polymer-BODIPY energy transfer, and the plot was compared to that recorded for the anionic membrane under the same conditions (Figure 2b). The fluorescence intensity of BODIPY increased abruptly within a few minutes after polymer addition, indicating that the interaction of HTMA-PFP with the zwitterionic lipid bilayer is very fast; however, it was slower than that observed for the anionic membrane, which occurred within the first few seconds.

**Figure 3.**
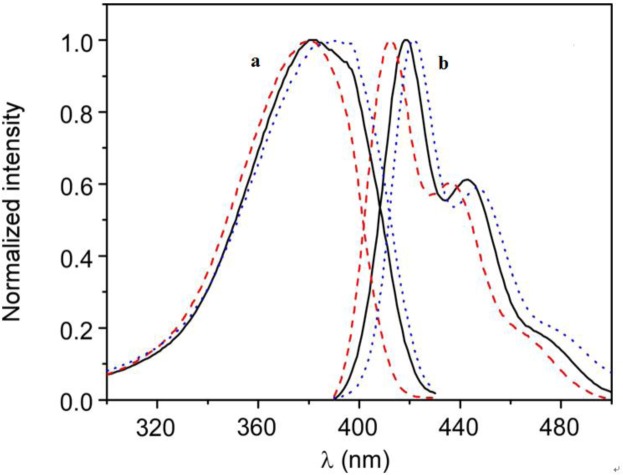
Normalized (**a**) excitation and (**b**) emission fluorescence spectra of HTMA-PFP in buffer (blue dotted line), LUVs of DMPC (black line) and LUVs of DMPG (red dashed line) recorded at 40 °C.

The higher affinity of the polymer for the anionic vesicles and faster incorporation kinetics indicate that the nature of the interaction between HTMA-PFP and the lipid membrane depends on the lipid charge, which is mainly electrostatic for the anionic system, at least in a first step, while for the zwitterionic one it would likely be mediated by hydrophobic interaction between the conjugated backbone of the polymer and the lipid molecules. The differences observed in the fluorescence spectra of the polyelectrolyte in both types of lipids also point to a different interaction mode. Figure 3 shows the normalized excitation and emission spectra of HTMA-PFP in DMPC as compared to those obtained in DMPG and buffer. In DMPG, the emission maximum intensity was observed at 411 nm, while in DMPC and buffer, it was located at 418 and 422 nm, respectively. Simultaneously to the blue-shift, an increase in fluorescence intensity was observed (data not shown), which probably indicates that the metastable aggregates formed in buffer break-up upon interaction with membranes; this effect was more pronounced in the anionic bilayer than in the zwitterionic. The break-up of aggregates would decrease the conjugation length, shifting the emission spectrum to shorter wavelengths. The excitation spectra support this interpretation, since the red-shift and broadness of the band are associated with an increase in the aggregation of the polyelectrolyte [20,25,41]. All these results make evident the different interaction mechanism and suggest that aggregates of HTMA-PFP are better solubilised in the anionic membrane than in the zwitterionic.

**Figure 4.**
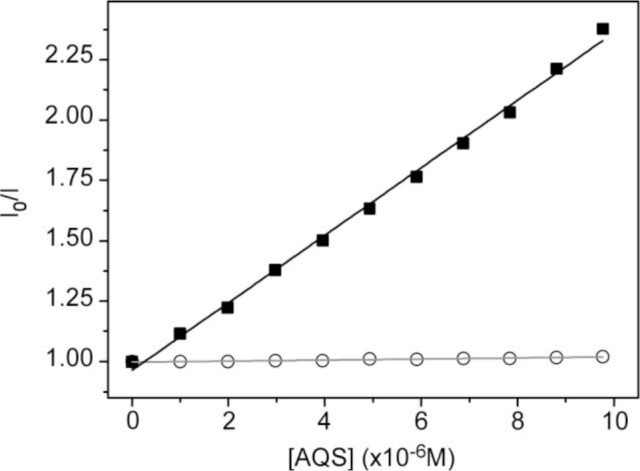
Stern-Volmer plots for quenching of HTMA-PFP (1.5 μM) by AQS in LUVs of DMPC (solid squares) and in LUVs of DMPG (circles). Measurements were performed at 40 °C.

**Figure 5.**
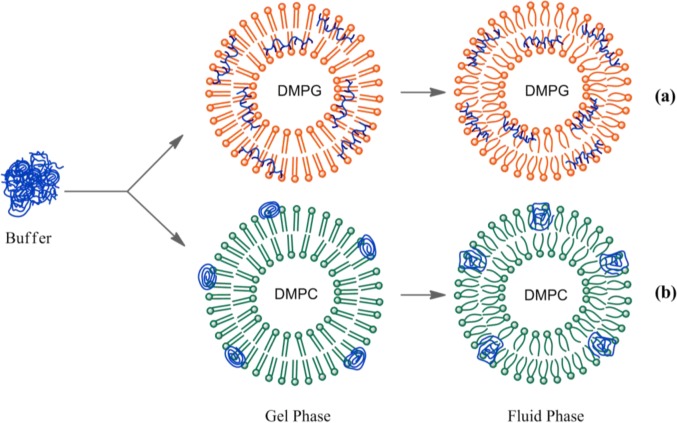
Schematic model, which shows the interaction between HTMA-PFP and lipid vesicles composed of (**a**) anionic and (**b**) zwitterionic vesicles in gel and fluid phases.

To obtain more insight of the membrane-polyelectrolyte interaction, quenching experiments were carried out using the anionic electron acceptor 9,10-anthraquinone-2,6-disulfonic acid (AQS) as a fluorescence quencher. This molecule has been reported to be an excellent quencher for cationic conjugated polyelectrolytes and is soluble in water, but not in lipid membranes [42,43]. In our previous paper, AQS was not capable of deactivating HTMA-PFP’s fluorescence when HTMA-PFP was incorporated into anionic DMPG vesicles, indicating that the polyelectrolyte does not remain located close to the membrane surface, but is embedded into the lipid bilayer [31]. A similar experiment was performed in zwitterionic vesicles and the results are shown in Figure 4. Contrary to what was observed for anionic membranes, when increasing concentrations of AQS were added to a suspension of DMPC LUVs containing HTMA-PFP, a decrease in the fluorescence signal of the polyelectrolyte was observed. The corresponding Stern-Volmer plot was linear in the concentration range studied (up to 10 μM) and a value of *K_sv_* = 1.4 × 10^5^ M^−1^ was extracted from the slope of the plot. This value was used to calculate the bimolecular rate constant of the quenching process *k_q_*, assuming *τ* = 520 ps [30]. A high value of 2.7 × 10^14^ M^−1^·s^−1^ was found, which indicates, as expected, the formation of static quenching complexes between AQS and polymer. This value is, however, lower than that of 1.5 × 10^16^ M^−1^·s^−1^ obtained in buffer [31], indicating a more reduced accessibility of the quencher to the membrane-bound polyelectrolyte as compared to the polyelectrolyte in solution. This result confirms that HTMA-PFP is incorporated into the lipid vesicle, but indicates that it remains near or at the surface of the bilayer and not in the hydrophobic core (see Figure 5).

**Figure 6.**
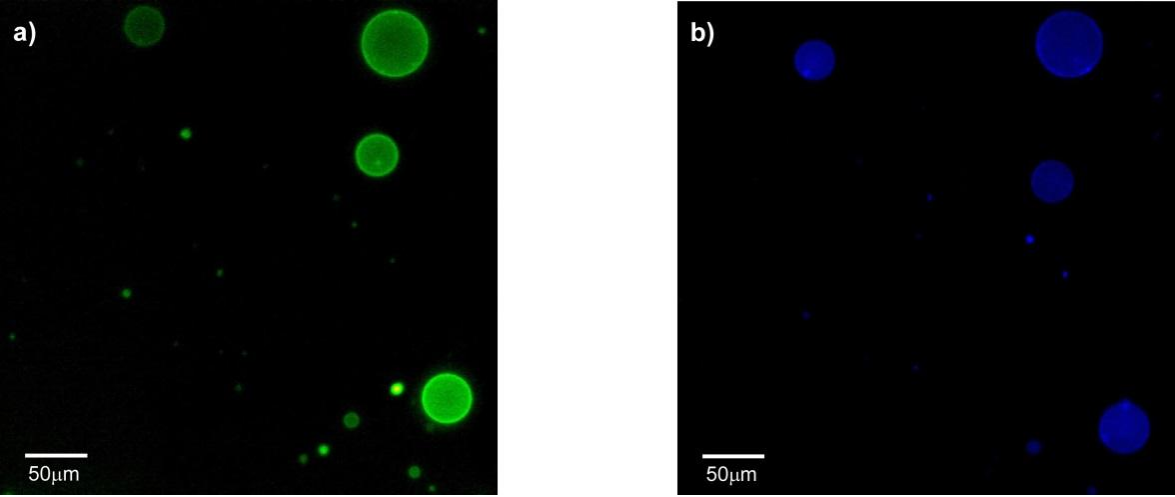
Fluorescence microscopy images of GUVs composed of DOPC labelled with BODIPY-PC, recorded upon irradiation with (**a**) visible- and (**b**) UV-light, after addition of HTMA-PFP.

HTMA-PFP’s interaction with the zwitterionic lipid membrane was also evaluated from fluorescence microscopy images, using giant unilamellar vesicles (GUVs) prepared by the electroformation method. GUVs were composed of the zwitterionic phospholipid 1,2-Dioleoyl-*sn*-glycero-3-phosphocholine (DOPC) instead of DMPC to facilitate vesicle formation. GUVs were labelled with the fluorophore BODIPY-PC, which absorbs in the visible range without interfering with the absorption of HTMA-PFP. Images were recorded at 25 °C upon irradiation with visible- and UV-light. Figure 6 shows representative images of vesicles obtained after addition of low polymer concentrations. The results confirmed that HTMA-PFP was incorporated into the lipid vesicles, retaining their structural integrity without altering their spherical morphology. No photobleaching was observed during the acquisition period, confirming the photostability of HTMA-PFP in the zwitterionic system and its possible utilization as a fluorescent membrane marker, as was already reported in the previous paper for anionic vesicles [31].

### 2.2. Effect of Temperature

It is well known that membranes composed of a single species of lipid molecules display a thermal phase transition associated with trans-gauche isomerizations of the lipid chains, which brings the bilayer from an ordered gel to a disordered fluid state. In biological membranes, the lipids are mainly in a fluid phase, which facilitates lateral diffusion and conformational changes of membrane proteins. Therefore, to better simulate biological membranes, all the aforementioned experiments were carried out at 40 °C to ensure that the lipid bilayer was in the fluid phase (both DMPC and DMPG present their thermal phase transition around 24 °C), with exception of the microscopy experiments which were performed at 25 °C, since the transition temperature of DOPC occurs below 0 °C. However, it is of interest to know how the large structural changes taking place in the lipid bilayer in the lipid phase transition affect polyelectrolyte behaviour. This study might lead to more insight into the polymer-membrane interaction mechanism. The effect of temperature on HTMA-PFP incorporated in DMPG was explored in the previous paper [31]. Results showed a clear red-shift in the emission spectrum when the membrane was in the gel phase and a slight decrease in fluorescence intensity accompanied by an increase in the vibrational structure. This behaviour was explained by the chemical structure of the polyelectrolyte shown in Figure 1. Probably, the tight packing of lipid chains below the transition temperature forces the polymer to adopt a planar conformation of phenyl rings, reducing its conformational flexibility and extending the effective π-conjugation length, shifting the emission to red [44]. In contrast, above the transition temperature, the polymer can twist to form various conformations, preventing the delocalization of π-electrons over the entire chain (Figure 5a).

**Figure 7.**
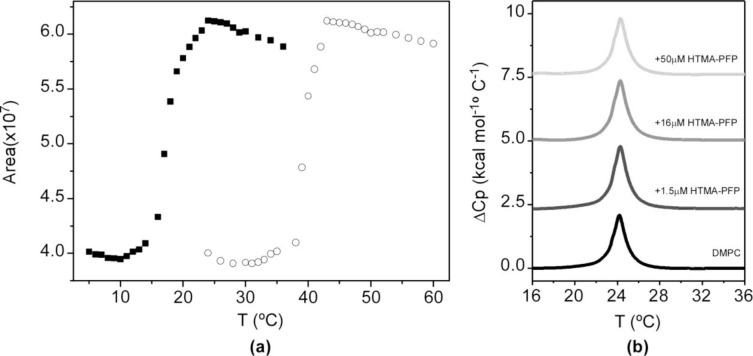
(**a**) Temperature dependence of the fluorescence intensity measured as the integrated area under the spectrum of HTMA-PFP (1.5 μM), recorded in DMPC (black squares) and DPPC (circles); (**b**) DSC thermograms of DMPC LUVs in the absence (*T_m_* = 24.5; *∆H* = 6.0 kcal/mol) and presence of HTMA-PFP at concentrations of 1.5 μM (*T_m_* = 24.5; *∆H* = 6.9 kcal/mol), 16 μM (*T_m_* = 24.5; *∆H* = 6.6 kcal/mol) and 50 μM (*T_m_* =24.5; *∆H* = 6.1 kcal/mol).

When the effect of temperature was explored in zwitterionic vesicles, a different behaviour was observed as compared with that in anionic vesicles. In this case, the fluorescence quantum yield of HTMA-PFP decreased abruptly in the gel phase and only a very slight red shift was detected in the emission spectrum (~2 nm). Changes in fluorescence intensity were plotted as a function of temperature (Figure 7a). The results showed that from 5 to 12 °C, the fluorescence signal was practically constant. However, a significant increase was observed from 13 °C, reaching its maximum at 24 °C. One of the possible explanations for this result is that the presence of the polymer in the bilayer disrupts the van der Waals interactions between phospholipids, shifting the phase transition to lower temperatures. To explore this possibility, differential scanning calorimetry (DSC) experiments were performed on DMPC LUVs containing HTMA-PFP. Figure 7b shows the thermograms recorded in the absence or presence of increasing concentrations of polyelectrolyte (1.5, 16 and 50 μM). From the analysis of these thermograms, transition temperatures (*T_m_*) and enthalpies were calculated (see legend in Figure 7b). The fact that the values obtained for DMPC containing polyelectrolyte were similar to those of the pure lipid, even at 50 μM of concentration, indicates that the cooperativity of the transition is preserved in the presence of the polymer and, therefore, HTMA-PFP is not disrupting the overall structure of the lipid bilayer at the used concentrations. The second possibility is that the polyelectrolyte is sensitive to the lipid pretransition. The lipid pretransition is a low enthalpy transition below the main phase transition of lipid membranes in which a flat membrane in the gel phase transforms into a periodically undulated bilayer (ripple phase) with a corrugated surface profile. Generally, it is assumed that the lipids in the ripple phase are mainly in the *all-trans* configuration, as in the gel phase. However, several studies point to the existence of fluid regions coupled with the geometry of the ripples and suggest that both pretransition and main transition are caused by the same physical effect, namely chain melting [45,46]. For DMPC, this phenomenon take places around 14 °C. Therefore, the increase in the fluorescence intensity of HTMA-PFP at 14 °C might be attributable to the conformational changes and/or to the change in location experienced by the polymer as a consequence of these structural alterations, which mainly affect the membrane surface. In order to check this possibility, we carried out a similar experiments, but incorporated the polyelectrolyte in lipid vesicles composed of 1,2-Dipalmitoyl-*sn*-glycero-3-phosphocholine (DPPC), a zwitterionic phospholipid that presents its pretransition and main phase transition at 33.5 and 41 °C, respectively. In this case, HTMA-PFP fluorescence intensity was practically constant up to 33 °C and increased from 34 to 42 °C, coinciding with the initiation and completion of the acyl chain melting process (Figure 7a). DSC analysis was also performed for DPPC in the absence and presence of different concentrations HTMA-PFP. As was observed for DMPC, the *T_m_* and transition enthalpies were similar to those of pure lipid (data not shown).

This result supports the hypothesis that HTMA-PFP aggregates are located near the bilayer surface and are, therefore, sensitive to changes occurring in this membrane region. Probably, below the pretransition, the polymer aggregates are mainly adsorbed on the surface of the vesicles instead of incorporated into the lipid bilayer (Figure 5). The beginning of pretransition allows the aggregates to solubilize better in the lipid membrane, leading to an increased fluorescence intensity. To confirm this assumption, a quenching experiment, similar to the one described before, was carried out in DMPC below the pretransition temperature at 10 °C. The quenching efficiencies were lower than those observed for the polymer in buffer, but 3-times higher than those in the fluid phase, suggesting a higher accessibility of the quencher to the polyelectrolyte (data not shown). This result indicates that, at this temperature, HTMA-PFP still interacts with the zwitterionic membrane, but remains in the lipid/water interface and is more exposed to the solvent than in the fluid phase.

### 2.3. Selectivity of HTMA-PFP against Anionic and Zwitterionic Membranes

**Figure 8.**
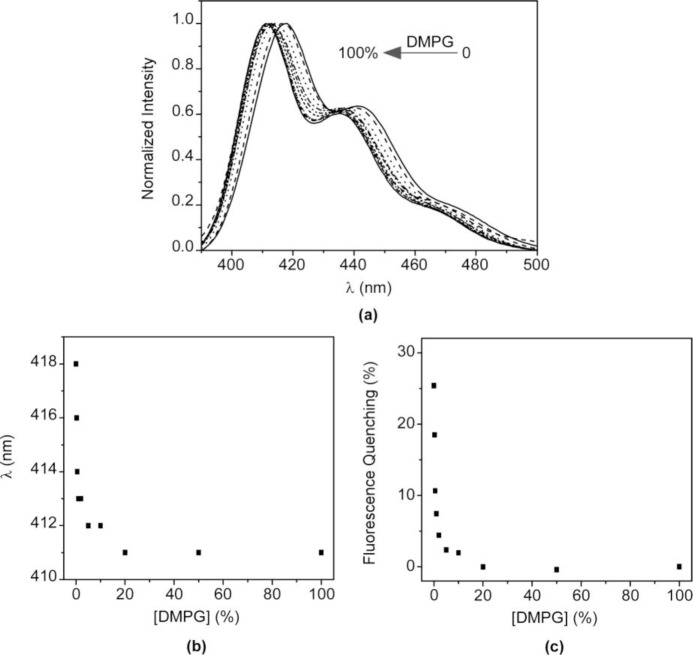
(**a**) Normalized fluorescence emission spectra of HTMA-PFP (1.5 μM) in a mixture of DMPC and DMPG LUVs (final lipid concentration 1 mM) recorded at increasing anionic vesicle content; (**b**) position of the fluorescence maximum as a function of increased DMPG vesicle percentage; (**c**) fluorescence quenching percentage of HTMA-PFP (1.5 μM) with AQS (5 μM) *versus* increased DMPG vesicle percentage.

The above results confirm that the nature of the interaction between HTMA-PFP and lipid membranes depends on the lipid charge. Although an electrostatic interaction is expected to be responsible for the high affinity of HTMA-PFP for the anionic membrane, the final location of the polyelectrolyte, which is well embedded into the bilayer core, indicates that hydrophobic forces also contribute to its solubilisation, reducing its aggregation state and increasing the fluorescence quantum yield compared to zwitterionic membranes. This behaviour suggests that HTMA-PFP is selective for anionic vesicles and can, therefore, be used as a potential bacterial membrane marker. The fact that the position of the fluorescence emission spectrum of HTMA-PFP depends on the lipid charge was used as a tool to confirm this hypothesis in this study. A series of samples containing different ratios of DMPC and DMPG LUVs (final concentration 1 mM) were prepared and the fluorescence spectrum of HTMA-PFP was recorded for each sample at 40 °C (Figure 8a). The results show a clear shift of the spectral maximum from 418 to 411 nm, when the DMPG LUVs content is increased. When the sample was composed of equal amounts of DMPC and DMPG, the spectral maximum was observed at 411 nm, indicating that all the polymer chains were incorporated into the anionic membrane and not into the zwitterionic. These results confirm the higher affinity of HTMA-PFP for the anionic membrane and can be better seen by plotting the wavelength of the maximum spectral as a function of the lipid composition (Figure 8b). The plot shows that the presence of a small amount of DMPG vesicles is sufficient to observe the position of the maximum closer to 411 than to 418 nm. This conclusion was supported by quenching experiments, which were carried out on the samples using AQS as a quencher (Figure 8c). If effectively all the polymer chains are embedded in the anionic vesicles, then they should not be accessible to the quencher. The fact that the quenching efficiency was close to 0% for samples containing a small amount of DMPG LUVs confirms this hypothesis, confirming the selectivity of HTMA-PFP for anionic lipids.

**Figure 9.**
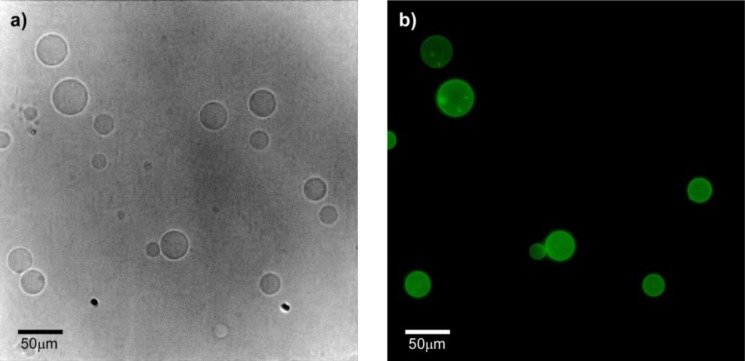
Microscopy images of a mixture of GUVs of DOPC-BODIPY and DOPC/DOPG (3:1) observed by (**a**) phase contrast microscopy and (**b**) upon excitation with Vis-light (both images correspond to the same field).

**Figure 10.**
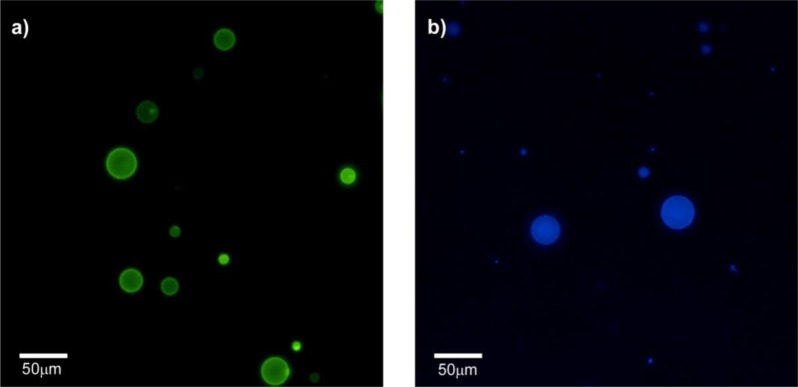
Fluorescence microscopy images of a mixture of GUVs of DOPC-BODIPY and DOPC/DOPG (3:1) observed after HTMA-PFP addition, upon excitation with (**a**) Vis-light and (**b**) UV-light (both images correspond to the same field).

The preference of HTMA-PFP towards anionic membrane suggests, but does not prove, that the polyelectrolyte can be used to label and visualize populations of anionic vesicles over zwitterionic vesicles. To test this ability, we prepared two series of GUVs at the same lipid concentration, but with different composition. The first GUVs were composed of zwitterionic lipids of DOPC and labeled with the fluorescent probe BODIPY-PC, which could be visualized upon excitation with visible-light. The second series of GUVs also contained DOPC, but were mixed with anionic lipids of 1,2-Dioleoyl-*sn*-glycero-3-phospho-rac-(1-glycerol) (DOPG) in a molar ratio of 3:1 (note that GUVs of pure anionic lipids cannot be formed by the electroformation method); no fluorescent probe was added, so they could not be observed via fluorescence microscopy. Equivalent volumes of both preparations were transferred to the same well and microscopy images were recorded before and after the addition of HTMA-PFP. As expected, in the absence of the polymer both types of vesicles were visualized by phase contrast microscopy (Figure 9a), but only some of them (the zwitterionic) were fluorescent under Vis-light (Figure 9b), and no fluorescence image was detected when the sample was excited with UV-light. Fluorescence microscopy images recorded after addition of polyelectrolyte are shown in Figure 10. The pictures show that the vesicles that fluoresce in green upon irradiation with visible light (Figure 10a) are different from those fluorescing in blue with UV-light (Figure 10b). This result is direct evidence that the polyelectrolyte selectively labels the anionic vesicles and, therefore, supports its potential use as a bacterial imaging probe.

### 2.4. Ability of HTMA-PFP to Destabilize Anionic Vesicles

**Figure 11.**
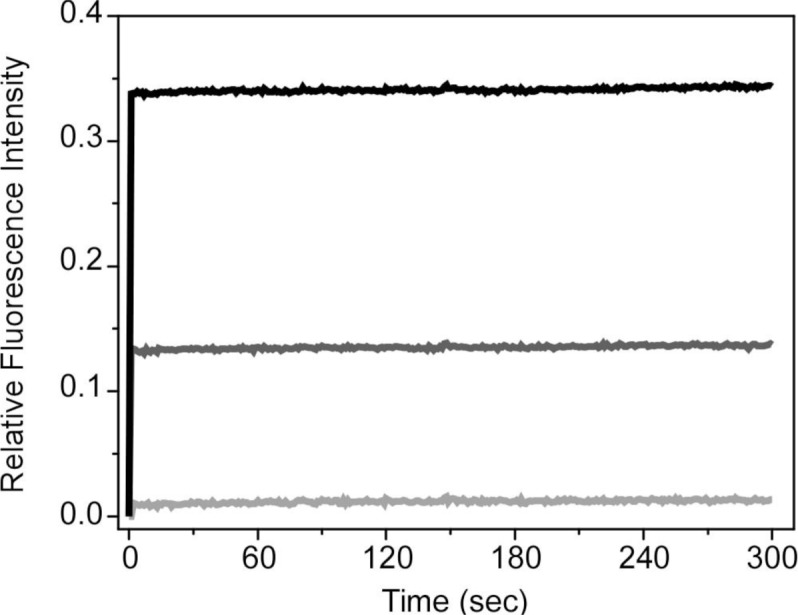
Fluorescence leakage profiles from DOPG vesicles with the addition of HTMA-PFP (1.5 μM; light grey, 22 μM; grey, 60 μM; black line) to phosphate buffer at room temperature.

Molecules with quaternary amine groups have been reported to disrupt bacterial membranes causing cell death [36,37]. The biocide action involves perturbation of the bacterial membrane lipid bilayers through interaction of the positively charged quaternary nitrogen with the polar phospholipid head groups. The hydrophobic part of the molecule subsequently interacts and inserts into the hydrophobic membrane core, causing the rearrangement of the membrane and the subsequent leakage of intracellular constituents [38]. Recently, a series of cationic poly(phenylene ethynylene) (PPE)-based conjugated polyelectrolytes containing pendant quaternary amine groups have been described as exhibiting biocidal activity against a variety of bacterial species [47]. In order to explore if HTMA-PFP with these cationic groups could also be used to this end, we evaluated the membrane stability of anionic vesicles as a function of time in the presence of increasing concentrations of polyelectrolyte. Stability was assessed through leakage experiments using the fluorophore carboxyfluorescein (CF), whose absorption and fluorescence do not interfere with that of HTMA-PFP. CF was encapsulated at high concentrations in the aqueous cavity of LUVs composed of DOPG, as described in the Experimental Section. Under these conditions, the CF fluorescence is very low due to self-quenching [48,49]. If the vesicle membrane is perturbed by the incorporation of polyelectrolyte, then the dye is released and the fluorescence signal increases after its dilution in buffer. CF leakage was calculated from Equation (3):

$$\text{CF leakage}=\frac{F-F_{0}}{F_{\text{max}}-F}$$ (3)

where *F*_0_ and *F* are the fluorescence intensities of CF in the vesicles in the absence and presence of HTMA-PFP, respectively, and *F*_max_ is the maximum fluorescence intensity of the sample observed after addition of Triton-X100 (10%) that causes the complete lysis of the vesicles. Figure 11 shows the fluorescence leakage profiles from DOPG vesicles upon addition of three increasing concentrations of polyelectrolyte. Results show that at 1.5 μM of HTMA-PFP, the fluorescence leakage was practically zero and therefore the integrity of the anionic vesicle is maintained at this polymer concentration, as was observed in the previous study [31]. However, higher concentrations caused membrane perturbation and the dye leakage increased as a function of the polymer concentration. The leakage process was very fast, occurring within the first few seconds after the addition of the polyelectrolyte. This suggests that, at these concentrations, the polymer is able to alter the integrity of the anionic vesicles, and that, once internalized, it quickly disrupts the lipid bilayer resulting in membrane rupture.

## 3. Experimental Section

### 3.1. Materials

The cationic CPE HTMA-PFP (*M* = 8340 g/mol, repeat unit molecular weight, 694.71 g/mol; *n*’ = 12 based on polyfluorene calibration) was obtained and characterized in our laboratory as previously described [50,51]. In brief, a low-molecular-weight batch of the neutral polymer, poly[9,9-bis(6’-bromohexyl)fluorene-phenylene], was synthesized by Suzuki coupling with Pd(II) as a catalyst and treated with gas-phase trimethylamine to obtain the corresponding cationic polyelectrolyte. Stock solutions of HTMA-PFP (3.65 × 10^−4^ M, in repeat units) were prepared in DMSO and stored at −20 °C before use. The fluorescent probes 5-butyl-4,4-difluoro-4-bora-3a,4a-diaza-s-indacene-3-nonanoic acid (BODIPY 500/510 C_4_,C_9_) and 2-(4,4-difluoro-5-methyl-4-bora-3a,4a-diaza-s-indacene-3-dodecanoyl)-1-hexadecanoyl-sn-glycero-3-phosphocholine (BODIPY-PC) were from Molecular Probes (Eugene, OR, USA). Stock solutions (1 mM) of these probes were prepared in ethanol and stored at −20 °C before use. The fluorescent probe 5(6)-carboxyfluorescein (CF) and the quencher 9,10-anthraquinone-2,6-disulfonic acid (AQS) were obtained from Sigma-Aldrich (St. Louis, MO, USA) and dissolved in DMSO (1.25 M) and water (5 mM), respectively, just before use. The synthetic phospholipids 1,2-Dioleoyl-sn-glycero-3-phosphocholine (DOPC), 1,2-Dioleoyl-sn-glycero-3-phospho-rac-(1-glycerol) sodium salt (DOPG), 1,2-Dimyristoyl-sn-glycero-3-phosphocholine (DMPC), 1,2-Dimyristoyl-sn-glycero-3-phospho-rac-(1-glycerol) sodium salt (DMPG) and 1,2-Dipalmitoyl-sn-glycero-3-phosphocholine (DPPC) were from Sigma-Aldrich and used as received. All other compounds were of analytical or spectroscopic reagent grade. Sodium phosphate buffer (50 mM, 0.1 M NaCl, pH 7.4) was prepared with water, which was twice distilled in all-glass apparatus and deionized using Milli-Q equipment (Millipore, Madrid, Spain).

### 3.2. LUVs Formation

Chloroform/methanol solutions containing 3 mg of total phospholipid (DOPC, DOPG, DMPC, DMPG, DPPC) were dried first by evaporation under dry nitrogen gas stream and subsequently under vacuum for 3 h. Multilamellar vesicles (MLVs) were formed by resuspending the dried phospholipids (0.5 mM for DOPG and DMPG, 1 mM for DOPC, DMPC and DPPC) in the buffer to the required final concentration. The vesicle suspension was then heated at a temperature above the phospholipid phase transition and vortexed several times. Large unilamellar vesicles (LUVs) were prepared from these MLVs by pressure extrusion through 0.1 μm polycarbonate filters (Nucleopore, Cambridge, MA, USA). For energy transfer experiments, the probe BODIPY C_4_ C_9_ (at the adequate probe-to-lipid ratio of 1:500) was initially added to the chloroform/methanol solutions containing DMPC and DMPG and LUVs were subsequently prepared as described above.

### 3.3. Preparation of Giant Unilamellar Vesicles (GUVs)

Giant unilamellar vesicles (GUVs) composed of DOPC/DOPG (3/1) and DOPC containing fluorescent probe, BODIPY-PC at the adequate probe-to-lipid ratio (1:250), were prepared by the electroformation method [52,53] using custom made Pt electrode-containing Teflon chambers. Briefly, 1.2 μL of a 1 mM lipid solution in chloroform containing the fluorescent probe was spread on each side of the Pt electrode. Removal of organic solvent traces was carried out by vacuum dehydration. Afterwards, the dried electrodes were hydrated with 450 μL of a 200 mM sucrose solution in Milli-Q purified water. Subsequently, 7 V voltage and 10 Hz frequency signal were applied for 2 h, followed by 1 Hz frequency signal for 30 min. After GUV formation, the sample was collected from the chambers and transferred to the wells of a micro-slide plastic plate, which deposits approximately 500 μL of preparation. To better observe the GUVs under the microscope, 400 μL of a 200 mM glucose solution were previously added to the wells in order to settle the 50 μL of GUVs to the bottom of the chamber. Samples were preserved for 2 h at 10 °C before microscopic visualization.

### 3.4. Preparation of HTMA-PFP/Lipid Samples

Aliquots of HTMA-PFP in DMSO were externally added to the lipid vesicle suspension, well above the phospholipid phase transition to ensure that the membrane was in the fluid phase. In all cases, the proportion of DMSO in the aqueous sample was always lower than 1% (V/V). In most samples, the final concentration of HTMA-PFP was 1.5 μM in terms of repeat units.

### 3.5. Absorption and Fluorescence Spectra

Absorption measurements were carried out at room temperature using a UV-1603 spectrophotometer (Shimadzu, Tokyo, Japan). Fluorescence spectra and fluorescence intensity measurements were performed on a QuantaMaster spectrofluorometer (PTI, Birmingham, NJ, USA) interfaced with a Peltier cell. The experimental samples were placed in 10 mm × 10 mm path length quartz cuvettes. The excitation wavelength for HTMA-PFP was set to 380 nm. Background intensities were always checked and subtracted from the sample when necessary.

### 3.6. Fluorescence Microscopy Measurements

Fluorescence microscopy images were recorded using a Nikon Eclipse TE2000-U (Melville, NY, USA) inverted microscope equipped with a Nikon Digital Sight DS-1QM/H and Nikon Digital Camera DXM1200. UV excitation (340 nm ≤ λ_ex_ ≤ 380 nm) and blue emission (435 nm ≤ λ_em_ ≤ 485 nm) was filtered using a DAPI filter cube. Visible excitation (465 nm ≤ λ_ex_ ≤ 495 nm) and green emission (515 nm ≤ λ_em_ ≤ 555 nm) was filtered using a FITR filter cube. Data acquisition was monitored successively by manually formatting and data processing with NIS-Elements AR 2.30 software.

### 3.7. Quenching Experiments

Fluorescence emission of HTMA-PFP was studied in the absence and presence of different concentrations of AQS in DMPC LUVs. Stern-Volmer analysis was applied to the fluorescence quenching data according to Equation (4):

$$\frac{I_{0}}{I}=1+K_{sv}[Q]$$ (4)

where *I*_0_ and *I* stand for the steady-state fluorescence intensities in the absence and presence of quencher, respectively, and [*Q*] is the quencher concentration. The significance of *K_SV_* depends on the nature of the quenching process: it may represent the association constant for complex formation or the rate of dynamic quenching (*K_SV_* = *k_q_*τ*_0_*); where *k_q_* is the bimolecular rate constant of the quenching process and τ_0_ is the lifetime of the biomolecule. For dynamic quenching (diffusion-controlled quenching), *k_q_* may be as high as 10^10^ M^−1^·s^−1^. If *k_q_* is greater than this value, it usually indicates static quenching via a complexation between the fluorophore and the quencher.

### 3.8. Measurements of Vesicle Leakage Induced by Polymers

DOPG LUVs were prepared with carboxyfluorescein (CF) trapped in the aqueous interior at a concentration of 40 mM, in a buffer containing 50 mM phosphate and 0.1 M NaCl, pH 7.4. The non-encapsulated CF was removed by gel filtration over a column packed with Sephadex G-75 (Pharmacia, Uppsala, Switzerland) eluted with buffer containing 50 mM phosphate, 0.1 M NaCl, pH 7.4. Self-quenching is expected at high concentrations of CF within the small volume of the vesicle interior. Membrane rupture (leakage) of intraliposomal CF was assayed by treating the probe-loaded vesicles with the appropriate amounts of HTMA-PFP. The total rupture of the vesicles and release of CF was performed with Triton X-100 10%.

### 3.9. Calorimetry

Differential scanning calorimetry (DSC) experiments were performed in a high-resolution Microcal MC-2 differential scanning microcalorimeter (GE Healthcare, Piscataway, NJ, USA) under a constant external pressure of 30 psi in order to avoid bubble formation. The excess heat capacity functions were analyzed using Origin 7.0 (Microcal Software). Differences in the heat capacity between the sample and the reference cell, which contained only buffer, were obtained by raising the temperature 1 °C/min over a range from 16 to 36 °C for the DMPC samples in the absence and presence of different concentrations of the HTMA-PFP. A series of three consecutive scans of the same sample were performed to ensure scan-to-scan reproducibility and reversibility.

### 3.10. Molecular Dynamics Simulations

The three-dimensional model of HTMA-PFP was first generated using ChemDraw 10.0 (CambridgeSoft), and then refined with Chem3D (CambridgeSoft) using the MM2 energy minimization procedure.

## 4. Conclusions

The interaction of the cationic polyfluorene HTMA-PFP with zwitterionic lipid bilayers was characterized and compared with the interaction reported for anionic bilayers in order to explore the possibility of using this polyelectrolyte as a bacterial imaging probe. Results indicate that HTMA-PFP at low concentrations associates spontaneously with both type of lipid vesicles, retaining their structural integrity without altering their spherical morphology. The polyelectrolyte shows higher affinity for anionic membranes than for zwitterionic membranes, as well as a different final membrane location. While in the anionic membrane, the polyelectrolyte is embedded within the lipid bilayer, in the zwitterionic system it remains close to the surface, forming aggregates that are sensitive to the physical state of the lipid bilayer. On the basis of these results, it is possible to conclude that the nature of the interaction between HTMA-PFP and lipid membranes depends on the lipid charge. Although an electrostatic interaction was expected to be responsible for the high affinity for anionic membranes, the final location of the polyelectrolyte, which is well-embedded in the bilayer core, indicates that hydrophobic forces also contribute to its solubilisation, reducing its aggregation state and increasing its fluoresecence quantum yield compared to zwitterionic membranes. Fluorescence microscopy images of a mixture of anionic and zwitterionic vesicles confirm that HTMA-PFP selectively labels the anionic vesicles. In addition, leakage experiments indicate that the addition of higher amounts of polyelectrolyte destabilizes the anionic lipid bilayer, producing membrane rupture. Therefore, in conclusion, this work supports the use of HTMA-PFP as a fluorescent marker in membrane studies and indicates that, given its different behaviour towards anionic and zwitterionic membranes, the polyelectrolyte could be used for selective recognition, imaging, and killing of bacteria over mammalian cells.

## References

[1] Kepczynski M., Jamroz D., Wytrwal M., Bednar J., Rzad E., Nowakowska M. (2012). Interactions of a hydrophobically modified polycation with zwitterionic lipid membranes. Langmuir.

[2] Parhamifar L., Larsen A.K., Hunter A.C., Andresen T.L., Moghimi S.M. (2010). Polycation cytotoxicity: A delicate matter for nucleic acid therapy—Focus on polyethylenimine. Soft Matter.

[3] Stadler B., Chandrawati R., Goldie K., Caruso F. (2009). Capsosomes: Subcompartmentalizing polyelectrolyte capsules using liposomes. Langmuir.

[4] Ding L., Chi E.Y., Schanze K.S., Lopez G.P., Whitten D.G. (2010). Insight into the mechanism of antimicrobial conjugated polyelectrolytes: Lipid headgroup charge and membrane fluidity effects. Langmuir.

[5] Eren T., Som A., Rennie J.R., Nelson C.F., Urgina Y., Nusslein K., Coughlin E.B., Tew G.N. (2008). Antibacterial and hemolytic activities of quaternary pyridinium functionalized polynorbornenes. Macromol. Chem. Phys.

[6] Thomas S.W., Joly G.D., Swager T.M. (2007). Chemical sensors based on amplifying fluorescent conjugated polymers. Chem. Rev.

[7] Pu K.Y., Cai L., Liu B. (2009). Design and synthesis of charge-transfer-based conjugated polyelectrolytes as multicolor light-up probes. Macromolecules.

[8] Liu Y., Ogawa K., Schanze K.S. (2009). Conjugated polyelectrolytes as fluorescent sensors. J. Photochem. Photobiol. C Photochem. Rev.

[9] Ngo A.T., Karam P., Cosa G. (2011). Conjugated polyelectrolyte-lipid interactions: Opportunities in biosensing. Pure Appl. Chem.

[10] Wang B., Yuan H., Zhu C., Yang Q., Lv F., Liu L., Wang S. (2012). Polymer-drug conjugates for intracellar molecule-targeted photoinduced inactivation of protein and growth inhibition of cancer cells. Sci. Rep.

[11] Wang L., Li H., Cao D. (2012). Conjugated polyelectrolytes: Synthesis and application in biomolecule detection. Curr. Org. Chem.

[12] Feng G., Ding D., Liu B. (2012). Fluorescence bioimaging with conjugated polyelectrolytes. Nanoscale.

[13] Kim I.B., Shin H., Garcia A.J., Bunz U.H.F. (2007). Use of a folate-PPE conjugate to image cancer cells *in vitro*. Bioconjug. Chem.

[14] McRae R.L., Phillips R.L., Kim I.B., Bunz U.H.F., Fahrni C.J. (2008). Molecular recognition based on low-affinity polyvalent interactions: Selective binding of a carboxylated polymer to fibronectin fibrils of live fibroblast cells. J. Am. Chem. Soc.

[15] Nilsson K.P.R., Hammarström P. (2008). Luminescent conjugated polymers: Illuminating the dark matters of biology and pathology. Adv. Mater.

[16] Pu K.Y., Liu B. (2011). Fluorescent conjugated polyelectrolytes for bioimaging. Adv. Funct. Mater.

[17] Tang Y., Liu Y., Cao A. (2013). Strategy for sensor based on fluorescence emission red shift of conjugated polymers: Applications in pH response and enzyme activity detection. Anal. Chem.

[18] Zhu C., Yang Q., Liu L., Wang S. (2011). A potent fluorescent probe for the detection of cell apoptosis. Chem. Commun.

[19] Zhu C., Liu L., Yang Q., Lv F., Wang S. (2012). Water-soluble conjugated polymers for imaging, diagnosis, and therapy. Chem. Rev.

[20] Monteserín M., Burrows H.D., Valente A.J.M., Lobo V.M.M., Mallavia R., Tapia M.J., García-Zubiri I.X., di Paolo R.E., Maçanita A.L. (2007). Modulating the emission intensity of poly-(9,9-bis(6’-N,N,N-trimethylammonium)hexyl)-fluorene phenylene bromide through interaction with sodium alkylsulfonate surfactants. J. Phys. Chem. B.

[21] Wang S., Bazan G.C. (2004). Solvent-dependent aggregation of a water-soluble poly(fluorene) controls energy transfer to chromophore-labeled DNA. Chem. Commun.

[22] Burrows H.D., Lobo V.M.M., Pina J., Ramos M.L., de Seixas Melo J., Valente A.J.M., Tapia M.J., Pradhan S., Scherf U. (2004). Fluorescence enhancement of the water-soluble poly{1,4-phenylene-[9,9-bis-(4-phenoxybutylsulfonate)]fluorene-2,7-diyl} copolymer in *n*-dodecylpentaoxyethylene glycol ether micelles. Macromolecules.

[23] Knaapila M., Almasy L., Garamus V.M., Pearson C., Pradhan S., Petty M.C., Scherf U., Burrows H.D., Monkman A.P. (2006). Solubilization of polyelectrolytic hairy-rod polyfluorene in aqueous solutions of nonionic surfactant. J. Phys. Chem. B.

[24] Monteserín M., Tapia M.J., Ribeiro A.C.F., Santos C.I.A.V., Valente A.J.M., Burrows H.D., Mallavia R., Nilsson M., Soderman O. (2010). Multicomponent interdiffusion and self-diffusion of the cationic poly{[9,9-bis(6’-N,N,N-trimethylammonium)hexyl]fluorene-phenylene} dibromide in a dimethyl sulfoxide + water solution. J. Chem. Eng. Data.

[25] Martínez-Tome M.J., Esquembre R., Mallavia R., Mateo C.R. (2010). Formation of complexes between the conjugated polyelectrolyte poly{[9,9-bis(6’-N,N,Ntrimethylammonium)hexyl]fluorene-phenylene} bromide (HTMA-PFP) and human serum albumin. Biomacromolecules.

[26] Monteserín M., Burrows H.D., Valente A.J.M., Mallavia R., di Paolo R.E., Maçanita A.L., Tapia M.J. (2009). Interaction between poly(9,9-bis(6’-N,N,N-trimethylammonium)hexyl)]fluorene phenylene) bromide and DNA as seen by spectroscopy, viscosity, and conductivity: Effect of molecular weights and DNA secondary structure. J. Phys. Chem. B.

[27] Tapia M.J., Montserín M., Valente A.J.M., Burrows H.D., Mallavia R. (2010). Binding of polynucleotides to conjugated polyelectrolytes and its applications in sensing. Adv. Colloid Interface Sci.

[28] Liu B., Wang S., Bazan G.C., Mikhailovsky A. (2003). Shape-adaptable water-soluble conjugated polymers. J. Am. Chem. Soc.

[29] Liu B., Bazan G.C. (2006). Optimization of the molecular orbital energies of conjugated polymers for optical amplification of fluorescent sensors. J. Am. Chem. Soc.

[30] Pinto S.M., Burrows H.D., Pereira M.M., Fonseca S.M., Dias F.B., Mallavia R., Tapia M.J. (2009). Singlet-singlet energy transfer in self-assembled systems of the cationic poly{9,9-bis[6’-N,N,N-trimethylammoniumhexyl]fluorene-co-1,4-phenylene} with oppositely charged porphyrins. J. Phys. Chem. B.

[31] Kahveci Z., Martínez-Tomé M.J., Mallavia R., Mateo C.R. (2013). Use of the conjugated polyelectrolyte poly{[9,9-bis(6’-N,N,N-trimethylammonium)hexyl]fluorene-phenylene} bromide (HTMA-PFP) as a fluorescent membrane marker. Biomacromolecules.

[32] Leevy W.M., Gammon S.T., Jiang H., Johnson J.R., Maxwell D.J., Jackson E.N., Marquez M., Piwnica-Worms D., Smith B.D. (2006). Optical imaging of bacterial infection in living mice using a fluorescent near-infrared molecular probe. J. Am. Chem. Soc.

[33] Leevy W.M., Gammon S.T., Johnson J.R., Lampkins A.J., Jiang H., Marquez M., Piwnica-Worms D., Suckow M.A., Smith B.D. (2008). Noninvasive optical imaging of Staphylococcus aureus bacterial infection in living mice using a bis-dipicolylamine-zinc(II) affinity group conjugated to a near-infrared fluorophore. Bioconjug. Chem.

[34] Sasser T.A., van Avermaete A.E., White A., Chapman S., Johnson J.R., van Avermaete T., Gammon S.T., Leevy W.M. (2013). Bacterial infection probes and imaging strategies in clinical nuclear medicine and preclinical molecular imaging. Curr. Top. Med. Chem.

[35] Liu C., Gu Y. (2013). Noninvasive optical imaging of staphylococcus aureus infection *in vivo* using an antimicrobial peptide fragment based near-infrared fluorescent probes. J. Innov. Opt. Health Sci.

[36] Laopaiboon L., Hall S.J., Smith R.N. (2002). The effect of a quaternary ammonium biocide on the performance and characteristics of laboratory-scale rotating biological contactors. J. Appl. Microbiol.

[37] Kenawy E.R., Worley S.D., Broughton R. (2007). The chemistry and applications of antimicrobial polymers: A state-of-the-art review. Biomacromolecules.

[38] McBain A.J., Ledder R.G., Moore L.E., Catrenich C.E., Gilbert P. (2004). Effects of quaternary-ammonium-based formulations on bacterial community dynamics and antimicrobial susceptibility. Appl. Environ. Microbiol.

[39] Coutinho A., Prieto M. (1995). Self-association of the polyene antibiotic nystatin in dipalmitoylphosphatidylcholine vesicles: A time-resolved fluorescence study. Biophys. J.

[40] Davenport L., Dale R.E., Bisby R.H., Cundall R.B. (1985). Transverse location of the fluorescent probe 1,6-diphenyl-1,3,5-hexatriene in model lipid bilayer membrane systems by resonance excitation energy transfer. Biochemistry.

[41] Al Attar H.A., Monkman A.P. (2007). Effect of surfactant on water-soluble conjugated polymer used in biosensor. J. Phys. Chem. B.

[42] Chemburu S., Ji E., Casana Y., Wu Y., Buranda T., Schanze K.S., Lopez G.P., Whitten D.G. (2008). Conjugated polyelectrolyte supported bead based assays for phospholipase A_2_ activity. J. Phys. Chem. B.

[43] Chen J., Dong W.F., Möhwald H., Krastev R. (2008). Amplified fluorescence quenching of self-assembled polyelectrolyte—Dye nanoparticles in aqueous solution. Chem. Mater.

[44] Levitus M., Schmieder K., Ricks H., Shimizu K.D., Bunz U.H.F., Garcia-Garibay M.A. (2001). Steps to demarcate the effects of chromophore aggregation and planarization in poly(phenyleneethynylene)s. 1. Rotationally interrupted conjugation in the excited states of 1,4-bis(phenylethynyl)benzene. J. Am. Chem. Soc.

[45] Heimburg T. (2000). A model for the lipid pretransition: Coupling of ripple formation with the chain-melting transition. Biophys. J.

[46] Riske K.A., Barroso R.P., Vequi-Suplicy C.C., Germano R., Henriques V.B., Lamy M.T. (2009). Lipid bilayer pre-transition as the beginning of the melting process. Biochim. Biophys. Acta.

[47] Wang Y., Tang Y., Zhou Z., Ji E., Lopez G.P., Chi E.Y., Schanze K.S., Whitten D.G. (2010). Membrane perturbation activity of cationic phenylene ethynylene oligomers and polymers: Selectivity against model bacterial and mammalian membranes. Langmuir.

[48] Bandyopadhyay P., Bandyopadhyay P., Regen S.L. (2002). An ion conductor that recognizes osmotically-stressed phospholipid bilayers. J. Am. Chem. Soc.

[49] Van Renswoude J., Hoekstra D. (1981). Cell-induced leakage of liposome contents. Biochemistry.

[50] Mallavia R., Martinez-Peréz D., Chmelka B.F., Bazan G.C. (2004). Blue fluorescent films based on poly-2,7-fluorene-phenylene derivatives. Bol. Soc. Esp. Ceram. Vidrio.

[51] Molina R., Gómez-Ruiz S., Montilla F., Salinas-Castillo A., Fernández-Arroyo S., del Mar Ramos M., Micol V., Mallavia R. (2009). Progress in the synthesis of poly(2,7-fluorene-alt-1,4-phenylene), PFP, via suzuki coupling. Macromolecules.

[52] Angelova M.I., Dimitrov D.S. (1986). Liposome electroformation. Faraday Discuss. Chem. Soc.

[53] Esquembre R., Pinto S.N., Poveda J.A., Prieto M., Mateo C.R. (2012). Immobilization and characterization of giant unilamellar vesicles (GUVs) within porous silica glasses. Soft Matter.

